# LAT Software Induced Savings on Medical Costs of Alcohol Addicts' Care - Results from a Matched-Pairs Case-Control Study

**DOI:** 10.1371/journal.pone.0111931

**Published:** 2014-11-07

**Authors:** Mihajlo Jakovljevic, Mirjana Jovanovic, Nemanja Rancic, Benjamin Vyssoki, Natasa Djordjevic

**Affiliations:** 1 Department of Pharmacology and Toxicology; The Faculty of Medical Sciences, University of Kragujevac, Kragujevac, Serbia; 2 Department of Psychiatry; The Faculty of Medical Sciences, University of Kragujevac, Kragujevac, Serbia; 3 Centre for Clinical Pharmacology; Medical Faculty Military Medical Academy, University of Defense, Belgrade, Serbia; 4 Department of Psychiatry and Psychotherapy; Medical University of Vienna, Vienna, Austria; 5 Department of Pharmacology and Toxicology; The Faculty of Medical Sciences, University of Kragujevac, Kragujevac, Serbia; University Hospital Oldenburg, Germany

## Abstract

Lesch Alcoholism Typology (LAT) is one of the most widely used clinical typologies of alcohol addiction. Study tested whether introduction of LAT software in clinical practice leaded to improved outcomes and reduced costs. Retrospective matched-pairs case-control cost comparison study was conducted at the Regional Addiction Center of the University Clinic in Serbia involving 250 patients during the four-year period. Mean relapse frequency followed by outpatient detoxification was 0.42±0.90 vs. 0.70±1.66 (LAT/non-LAT; p = 0.267). Adding relapses after inpatient treatment total mean-number of relapses per patient was 0.70±1.74 vs. 0.97±1.89 (LAT/non-LAT; p = 0.201). However, these relapse frequency differentials were not statistically significant. Total hospital costs of Psychiatry clinic based non-LAT addicts' care (€54,660) were significantly reduced to €36,569 after initiation of LAT. Mean total cost per patient was reduced almost by half after initiation of LAT based treatment: €331±381 vs. €626±795 (LAT/non-LAT; p = 0.001). Mean cost of single psychiatry clinic admission among non-LAT treatment group was €320±330 (CI 95% 262–378) and among LAT €197±165 (CI 95% 168–226) (p = 0.019). Mean LAT software induced net savings on psychiatric care costs were €144 per patient. Total net savings on hospital care including F10 associated somatic co-morbidities amounted to €295 per patient. More sensitive diagnostic assessment and sub-type specific pharmacotherapy and psychotherapy following implementation of LAT software lead to significant savings on costs of hospital care.

## Introduction

Due to its high prevalence [1] and chronic clinical course [2], alcohol dependence represents a heavy burden for health care systems across the globe [3]. National health budgets are becoming increasingly constrained and therefore effortis put into assessment of cost-effectiveness of specificmedical treatment strategies, such as addiction specific psychotherapy techniques [4], [5] or anticraving medication for relapse prevention [6]–[8]. Theseresults contributed to more efficient care and evidence based allocation of resources [9]. However, the impactof distinct medical typologies that contain clinical practice guidelines on medical costs have not been researched thoroughly [10]–[13]. There are several typologies of alcohol dependence [14]–[16] which are widely adopted in the published literature.

Through the course of past century over a dozen of distinct clinical typologies of alcoholic dependence have been developed with Cloninger, Jellinek's, Babor's, LAT being only some of the most famous and broadly adopted. Lesch Alcoholism Typology (LAT), introduced in 1991, is regarded particularly helpful in terms of providing treatment recommendations grounded in patient's age of onset, family history, co-morbidity, and alcohol-related disabilities. The LAT typology is applied in clinical practice as a clinician's oriented software. It has been translated in 12 languages and validated in many countries, offering essential recommendations for pharmacological treatment of withdrawal and relapse prevention [17]–[21]. As to our best knowledge, so far there have been no attempts to assess the impact of clinical typologies implementation to the overall costs of alcohol addicts' medical care in a clinical trial. In this study we therefore want to evaluate the impact of a newly introduced sub-type specific treatment program, the LAT, on the cost of medical treatment in alcohol dependent patients.

## Materials and Methods

### Ethics Statement

The study was conducted according to Helsinki Declaration; current positive legislature on good clinical practice and guidelines on retrospective database analysis. Ethical committee approval was granted by the Ethical Committee of University Clinical Center Kragujevac, Serbia – Submission N° 01/11639 date 30. October 2013; Decision reached on 18. November 2013; N° 01-12299. Patient data were handled anonymously during the study. Patient records/information was anonymized and de-identified prior to analysis.

### Setting

Since January 2012, LAT typology and software, translated and administered in Serbian language, was introduced to the clinical practice in seven regional addiction centers throughout Serbia [22], [23]. The Regional Addiction Center Kragujevac provides services for up to 400 alcohol dependent patients per year and belongs to the Psychiatry Clinic of the single largest tertiary care university hospital in Central Serbia region with 1300 beds capacity (www.kc-kg.rs). Southeastern European country of Serbia has an upper middle income post-socialist transitional economy [9]. It's healthcare system is marked with typical strengths and weaknesses familiar to the most Eastern European economies including post-2004 EU members [22], [23]. Therefore, Serbia can be regarded representative of a wider regional setting. Its substantial national deficit of budget allocation to combat huge alcohol dependency burden emphasizes urgent need for effective resource allocation in this field [24]. So far, health economics principles were not part of routine policy making on reimbursement of antic raving medicines, detoxification procedures or psychotherapy techniques indicated in alcohol addiction treatment [25].

### Data

Study sample was selected out of 813 alcohol dependent cases being either outpatients or inpatients within the institutional framework of the Regional Addiction Center of the University Clinical Center Kragujevac, Serbia during the period 2010–2013. One hundred and twenty five case-control pairs were selected based on their clinically confirmed diagnosis of alcohol dependence according to ICD-10 and DSM-IV-TR, age and gender. There were no other grounds for patient selection except the possibility of close clinical matching so this was the maximum of available patient couples for comparison. LAT patients' medical services utilization was observed during 2012–2013 period, while non-LAT processed alcohol addicts were observedin2010–2011 period. Economic data on resource use and costs were extracted out of the University Clinical Center electronic registry of discharge invoices. Observed clinical setting was state owned not-for-profit hospital which is generally charging governmental budget subsidized prices to the citizens. Therefore this database presents reliable insight into the actual costs of care. Although individual periods while these costs accrued slightly varied, complete symmetry was obeyed in duration of the observation among cases coupled. That means that medical and resource use history for each single case was extracted either for one or for two years (depending on his/her actual physician visits and admissions) duration while his/her counterpart from control pool, was observed during equally long period of time (either one or two years). Clinical outcomes data accessible was individual frequency of relapse events followed by either outpatient detoxification or hospital admission. According to availability these relapses were evidenced for 2012–2013 observation time, in equal duration between patient pairs. Resource use and costs accrued due to alcohol dependence diagnostics, treatment and rehabilitation were observed separately within institutional frameworks of Psychiatry Clinic, Internal Medicine Clinic and Neurology Clinic for each case.

### Analysis

It is rather difficult to assess trends in clinical decision making and resource use patterns among alcohol addicts due to high clinical heterogeneity of patient's backgrounds [26]. Therefore, in the present study, matched-pairs case control retrospective study design was employed in a cost comparison analysis framework [27]. This approach, previously applied in clinical trials on addiction disorders [26]–[29], allows reliable elimination of statistical bias impact to the study results. Grounds for choosing such methodology was lying in the fact that medical services utilization patterns and associated costs otherwise might have been affected with differences between groups in clinical background, disease course and treatment response [30].

Costs were converted from national currency – Republic of Serbian Dinar (RSD) to Euro (€) in line with mean official exchange rates of the National Bank of Serbia in the respective years (www.nbs.rs). This way the effect of slight devaluation of domestic currency was avoided. There were no sudden swifts in pricing policy or significant changes in medical service charges related to alcoholism treatment during this period of time. Therefore chronological gap between experimental and control group (before and after LAT intervention) had rather minor impact to the price levels 2010–2013. Foreign currency (€) based prices remained stable.

### Statistics

Complete statistical analysis was carried out using Statistical Software PASW Statistics 18 and Microsoft Office Excel 2007. Cases and controls were matched according to sex, age and diagnosis. After pairing patients, cost analysis has done, during one-year or two-year period, between controls observed before and cases observed after implementation of LAT software - guidelines. Categorical variables were presented as frequencies of certain categories, while statistical significance of differences was tested using Chi-square test. Continuous variables were summarized as mean, standard deviation, range and 95% confidence interval. Continuous variables were compared using the Student's *t* test for independent samples and the Mann-Whitney U test, while nonparametric Kruskal Wallis H test was used to test the statistical significance among continuous variables with three or more categories. Analysis of money spending was done by non-parametric tests (the large standard deviations). Multiple regression analysis was performed on both LAT and non-LAT patients, while dependent variable were total hospital costs. Two models (LAT and non-LAT) were developed indicating key predictors of costs of care. All the analyses were estimated at p<0.05 level of the statistical significance.

## Results

### Number of relapses

Mean number of relapses followed by outpatient detoxification was 0.42±0.90 vs. 0.70±1.66 (LAT/non-LAT) (p = 0.267) (see Table 1). Frequency of relapses followed by hospital admission was 0.28±1.10 vs. 0.26±0.58 (LAT/non-LAT) (p = 0.339). Thus we come to the average rate of total relapses of 0.70±1.74 vs. 0.97±1.89 (LAT/non-LAT) while this difference was not statistically significant (p = 0.201).

**Table 1:** Demography and resource use patterns among alcohol dependent matched patient pairs, LAT vs. non-LAT comparison.

	LAT (n = 125)	Non-LAT (n = 125)	p value**
ICD-10 codes#	F.10.0–F10.9	F.10.0–F10.9	N/A
Age (M ± SD)	48.26±10.51	48.20±10.49	p = 0.966
Gender (male/female)	120/5	120/5	N/A
Relapses followed by hospital admission	0.28±1.10	0.26±0.58	p = 0.339
Relapses followed by outpatient detoxification	0.42±0.90	0.70±1.66	p = 0.267
Total relapse rates (2012–2013)	0.70±1.74	0.97±1.89	p = 0.201
Outpatient physician consultations	10.45±9.09	10.29±12.50	**p = 0.050** *
Inpatient physician consultations	7.19±57.81	2.95±9.37	p = 0.777
Mean N° Hospital admissions per patient - Psychiatry Clinic	1.24±1.42	1.25±1.42	p = 0.986
Hospital admissions N° - Internal Medicine Clinic (due to F10)	0.05±0.31	0.21±0.65	**p = 0.008** *
Hospital admissions N° - Neurology clinic (due to F10)	0.04±0.20	0.05±0.33	p = 0.491
Mean admissions N° (Internal + Neurology)	1.44±1.73	1.59±1.54	p = 0.151
Mean N° of all hospital admissions (M ± SD)	0.94±1.27	0.92±0.74	p = 0.855
Duration of Hospital admissions (M ± SD)	12.49±14.77	10.80±11.52	p = 0.315
Total hospital admissions – Psychiatry	155	156	N/A
Total N° of all hospital admissions – Internal Clinic	6	26	**p = 0.0016** *
Total N° of all hospital admissions – Neurology Clinic	5	6	p = 0.8415
Total joint Internal Medicine Clinic and Neurology Clinic hospital admissions	11	32	**p = 0.0047** *
Typology according to Lesch (type I/II/III/IV)	10/27/57/31	N/A	N/A

# All resource use at the Internal Medicine Clinic and Neurology Clinic were accounted exclusively for medical services consumed in relation to alcohol dependence diagnostics, treatment and rehabilitation (ICD-10 F10 diagnostic code group); p value- statistical signifiance of difference;

**- Mann-Whitney U test; !- Chi square test;

*- p<0.05; M ± SD- mean ± standard deviation.

### Inpatient and outpatient care

Total number of Psychiatry Clinic hospital admissions was 155 vs. 156 while there were 6 vs. 26 Internal Medicine Clinic admissions (Chi square test; p = 0.0016) and 5 vs. 6 Neurology Clinic admissions (Chi square test; p = 0.8415) (LAT/non-LAT) (see Table 1). Total joint Internal Medicine Clinic and Neurology Clinic hospital admissions frequency was 11 vs. 32 (LAT/non-LAT) (Chi square test; p = 0.0047). Mean Internal Medicine Clinic admissions frequency was significantly higher in non-LAT group 0.05±0.31 vs. 0.21±0.65 (p = 0.008).

**Figure 1:**
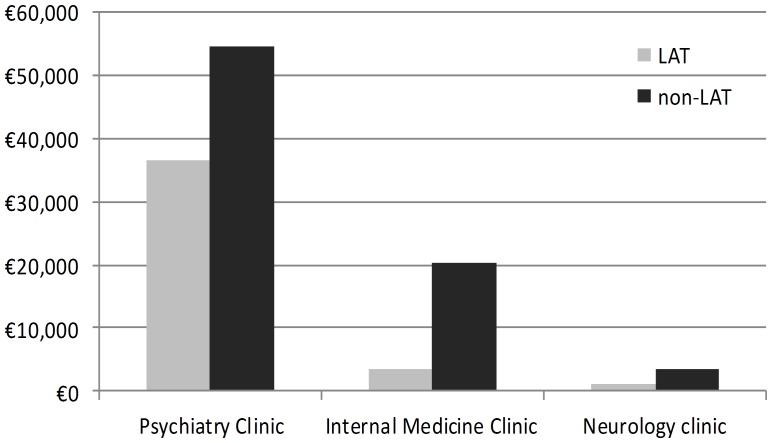
Costs of hospital care, inter-clinic differentials; total amounts per group; comparison LAT vs. non-LAT matched pairs (Y axis presents € values).

Mean hospital costs per patient due to joint Internal Medicine Clinic and Neurology Clinic admissions were higher among non-LAT patients €38±238 vs. €189±623 (LAT/non-LAT; p = 0.102) (see Table 2). Total costs of hospital care in all clinics were substantially lower in LAT group, €41,331 compared to €78,226 in non-LAT patients (p = 0.001) (see Table 2 and Fig. 1). Average net savings on direct medical costs of psychiatric hospital care highly likely attributable to the LAT software implementation were €144 per patient. Average net savings in total costs of hospital care for F10 associated mental and somatic disorders across all clinics were €295 per patient.

**Table 2:** Costs of hospital medical care; LAT vs. non-LAT comparison (€).

Cost domain (total values per group)#	LAT (n = 125)	Non-LAT (n = 125)	p value**
**Psychiatry Clinic**			
Outpatient Care	11,113	12,598	p = 0.773
Inpatient Care	25,456	42,062	**p = 0.008** *
Total costs of hospital care	36,569	54,660	**p = 0.008** *
**Main Cost Domains**			
**Physician Consultations**	6,132	7,942	p = 0.966
Outpatient physician consultations	5,074	4,785	p = 0.382
Inpatient physician consultations	1,058	3,157	p = 0.757
**Psychotherapeutic Interventions**	5,726	7,235	p = 0.720
**Hospital admission**	18,604	18,765	p = 0.430
**General Medical Care**	929	5,438	**p<0.001** *
**Laboratory Analysis**	2,044	6,794	**p<0.001** *
**Imaging Diagnostics**	887	2,976	**p = 0.044** *
**Pharmaceuticals**	2,246	5,510	p = 0.081
**Other Clinics**			
Internal Medicine Clinic	3,562	20,161	**p = 0.008** *
Neurology clinic	1,200	3,405	p = 0.497
Total somatic ward costs (Internal + Neurology Clinic)	4,762	23,566	p = 0.102
**Total Hospital costs**	**41,331**	**78,226**	**p = 0.001** *
Mean cost per patient – Psychiatry (M ± SD)	293±308	437±415	**p = 0.008** *
Mean cost per patient - Somatic wards/clinics (M ± SD)	38±238	189±623	p = 0.102
**Mean total hospital costs per patient** (M ± SD)	**331±381**	**626±795**	**p = 0.001** *

# All admission costs to the Internal Medicine Clinic and Neurology Clinic were accounted exclusively for medical services consumed in relation to alcohol dependence diagnostics, treatment and rehabilitation (ICD-10 F10 diagnostic code group); p value- statistical signifiance of difference;

**- Mann-Whitney U test;

*- p<0.05; M ± SD**-** mean ± standard deviation.

**Figure 2:**
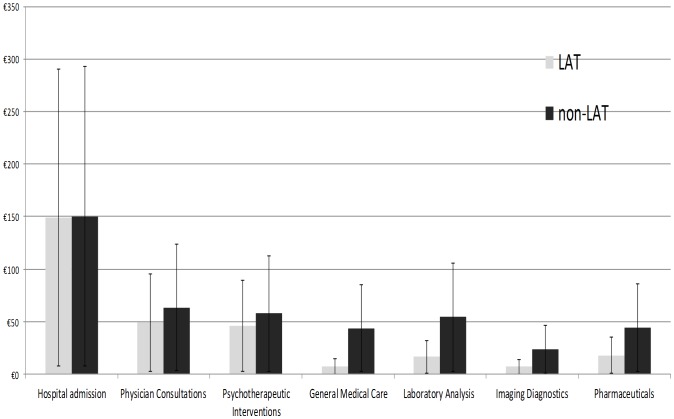
Cost domains; Mean values per patient presented with CI 95% range; comparison LAT vs. non-LAT matched pairs (Y axis presents € values).

Mean number of hospital admissions per patient to the Psychiatry Clinic, during the examined time period was 1.24±1.42/1.25±1.42 (LAT/non-LAT) (p = 0.986). Mean total duration of hospital admissions was 12.49±14.77/10.80±11.52 days (LAT/non-LAT) (p = 0.315). Attending psychiatrists delivered on average 10.69±9.06/12.81±16.06 sessions of social therapy, including implementation of psychotherapeutic techniques (LAT/non-LAT) (p = 0.200). Total Psychiatry Clinic hospital costs of non-LAT addicts' care amounted to €54,660, which were significantly reduced (€36,569, p = 0.008) after initiation of LAT (see Fig. 1). Mean cost of psychiatric care per patient decreased after initiation of LAT based treatment (€293±308 (CI 95%- 239-347) *vs*. €437±415 (CI 95%- 364-510) €), and the decrease was significant (p = 0.008). Mean per patient values of all cost domains in the non-LAT controls were higher than among LAT cases but only some of these findings were statistically significant (see Table 2; Fig. 2).

### Lesch Alcoholism Typology- Subtypes

Mean costs of care per LAT subtypes differed significantly (Kruskal Wallis H test; p = 0.005) and amounted to: LAT I – €326±88 (CI 95% 271–380) €, LAT II – €256±304 (CI 95% 141–370) €, LAT III – €256±295 (CI 95% 180–333) € and LAT IV – €381±368 (CI 95% 251–511) € among LAT classified patients. Mean cost of single hospital admission among non-LAT treatment group was €320±330 (CI 95% 262–378) € and among LAT €197±165 (CI 95% 168–226) € (Mann-Whitney U test; p = 0.019).

Multiple regression analysis was conducted using value of total hospital cost as dependent variable. It showed that model developed for LAT treated patients significantly better explaines costs variance compared to non-LAT (R^2^ = 0.517 vs. R^2^ = 0.132) (see Table 3 and Table 4). Variables exposing strongest impact to total cost of care among LAT cases were: age, duration of hospital admissions and outpatient physician consultations, while among non-LAT controls there were no such predictors with significant impact to the total cost of care.

**Table 3:** The impact of independent variables on total hospital cost among non-LAT treated patients (multiple regression analysis).

Model non-LAT	b	SE-b	beta	Pearson r
Constant	58.480	535.195		
Age	5.523	6.812	0.073	0.048
Gender	−15.258	328.707	−0.004	−0.007
Number of hospital admissions	265.450	140.739	0.246	0.316
Duration of hospital admissions	4.429	9.620	0.064	0.296
Outpatient physician consultations	−1.858	5.545	−0.029	−0.016
Inpatient physician consultations	11.342	8.243	0.134	0.211
Relapses followed by hospital admission	65.125	123.029	0.048	0.035
Relapses followed by outpatient detoxification	−9.298	43.931	−0.019	−0.066

Note: The dependent variable was Total hospital cost. R^2^ = 0.132, Adjusted R^2^ = 0.072.

**Table 4:** The impact of independent variables on total hospital cost among LAT treated patients (multiple regression analysis).

Model LAT	b	SE-b	beta	Pearson r
Constant	442.672	213.732		
Age*	−4.862	2.445	**−0.134**	−0.206
Gender	−94.649	130.726	−0.049	−0.109
Number of hospital admissions	66.641	40.408	0.221	0.645
Duration of hospital admissions*	11.905	3.450	**0.461**	0.675
Type of LAT	−16.626	29.024	−0.038	0.036
Outpatient physician consultations*	5.608	2.798	**0.134**	0.138
Inpatient physician consultations	0.233	0.442	0.035	0.159
Relapses followed by hospital admission	28.232	27.235	0.081	0.027
Relapses followed by outpatient detoxification	−26.307	33.150	−0.062	−0.087

Note: The dependent variable was Total hospital cost. R^2^ = 0.517, Adjusted R^2^ = 0.480; p<0.05*.

## Discussion

The impact of good clinical practice guidelines implementation to the clinicians' prescription patterns and their economic awareness was previously assessed and reported in recent studies [24]. Our study is the first to evaluate the impact of a newly introduced, sub-type specific alcohol dependency classification system on economic and clinical outcomes.

LAT cases exposed statistically significantly higher demand for outpatient physician consultations. On the opposite, Internal Medicine Clinic admissions were significantly more frequent among non-LAT controls. All other resource use differentials between the two groups were not statistically significant. With regards to costs of care, statistical significance in favor of LAT cases (substantially lower cost of care) was proved on numerous cost domains: Inpatient Care, Total costs of hospital care and Internal Medicine Clinic costs. Besides these aforementioned domains, LAT cases exhibited significantly lower costs of care compared to non-LAT controls in terms of mean cost per patient treated at Psychiatry Clinic as well as ultimately total hospital costs per patient. Noticed cost saving trend after LAT software and guidelines implementation in clinical practice was also statistically significant in consecutive domains: Laboratory analysis and Imaging diagnostics and General Medical (dominantly nursing) Care. An explanation for this phenomenon was overutilization of CTs and NMR imaging of the brain which was wide spreaded clinicans' practice before LAT introduction. Demands to perform blood tests covering too broad variety of targeted medical conditions (substance addiction, infections etc.) were also more frequent in 2010–2011. Cost savings on nursing care among LAT patients are partly attributable to more frequent outpatient visits to the attending psychiatrists recommended by LAT software acting as substitute for more costly hospital treatment being quite effective in controlling disease.

Resource use patterns exhibited in this trial point to the opportunity of reshaping clinician's behavior with evidence based guidelines [32]. We could observe frequency of outpatient visits to the attending psychiatrist and frequency of hospital admissions as an indirect sign of occasional relapses that these patients commonly experience [25].

LAT patients tended to consume less nursing care, laboratory analysis, imaging diagnostics and pharmaceuticals (see Table 2 and Fig. 2). Interestingly, recently published trial testing efficiency of sodium oxibate in maintaining alcohol abstinence among addicts showed that anticraving medicines could prove themselves useful regardless of actual LAT type of patient. This finding might support lack of significant differences between LAT and non-LAT cases in drug acquisition costs [31]. Lower demand for diagnostic services by the attending psychiatrists is attributable to the precise and focused clinical tools provided by interactive LAT software. These are allowing for earlier diagnostic recognition of patient type based on accurate anamnesis and examination criteria.

Statistically significant cost differentials among four stages of alcoholism clinical evolution according to LAT confirms broadly recognized LAT sensitivity for distinct recognition of patients with profoundly different drinking patterns and treatment needs [16]–[18], [21].

Non-LAT cases were more frequently treated at the Internal Clinic (Gastrointestinal and Hepatology wards) and Neurology Clinic due to alcohol related somatic disorders such as liver cirrhosis, polyneuritis and others. These wards demand much more expensive imaging examinations and blood tests compared to Psychiatry clinic [33]. Cost-reduction observed among LAT patients, probably happened due to their less severe somatic complications compared to respective non-LAT couples during the investigated time period consecutively somatic disorders worsened by occasional heavy drinking episodes were rather seldom among LAT cases. Authors believe that patient rambling among specialty Internal Medicine Clinic and Neurology Clinic delayed alcoholism type recognition and was additional underlying reason for their more demanding and expensive hospital care.

The hot issue of cost containment remains high on the agenda of regional health policy makers due to Western Balkan's difficulties in provision of sustainable health care funding, triggered by worldwide economic crisis [22], [23]. Cost saving procedures are considered by many experts a trade off with quality of medical services [34], [35]. In this particular alcohol addicted population, authors assumed that patients experienced clinical improvement because of more efficient diagnostic algorithm leading to the more targeted, individually adjusted treatment and weaker demand for health care. As a consequence, it appears that they needed less frequent outpatient physician consultations and consumed less resources which is the phenomena well described in literature [30]. Range of costs reported in upper-middle income Serbia is rather modest compared to high-income settings [24], [25]. Regardless of some evidenced intra-European differences in LAT sub-type structure across ethnicities [36], LAT associated savings should be expected elsewhere. Observed resource use patterns will likely cause decrease in overall costs of medical care for somatic symptoms, in other countries as well.

### Limitations

Matching of patients into couples with highly similar clinical background should have eliminated the potential bias and external validity issues in the study, which might have been affected by medical heterogeneity of samples in other similar clinical trials [26]. Regardless of few minor setbacks in this study design it provides high statistical reliability for generalizations of conclusions [37].

University Clinical Center Kragujevac electronic database of discharge invoices was used as a source of data on medical services utilization costs. Tight internal and external control of accounting offices of respective Clinics is conducted due to the fact that these invoices serve as grounds for negotiations on reimbursement with republican Health Insurance fund representatives. Therefore, these data should be regarded of satisfactory reliability and quality. Sample size is rather modest, due to the fact that authors had no access to other addiction centers throughout the country or abroad. According to authors' opinion this would probably be the core weakness of the study. Nevertheless, matching of patient pairs forms an appropriate control group allowing for the generalizability of conclusions. Deeper insight into indirect costs of lost productivity would be very helpful and would allow for more inclusive insight into the costs of care [38], as, due to adopting payer's perspective, these were rather out of scope of present trial. Clinical background data relied on relapse rates only. In real world clinical setting, there is no efficient way to assess any relapses that did not lead to additional treatment, so frequency of relapses marked as “total relapse rates” in this article, actually presents clinically visible fraction of them. Lack of an in-depth assessment of other clinical outcomes such as abstinence duration, was the another key weakness of this trial. The prospective approach would certainly provide deeper insight into the clinical efficiency of LAT based treatments.

## Conclusions

In conclusion, the significant medical savings documented by this matched-pair case-control study are anticipated consequence of more targeted therapeutic approach. Here we explain that noticed cost savings induced by LAT typology, and LAT software introduction the core underlying cost driver was higher rate of hospital admissions to the Internal Medicine wards due to severe somatic symptoms among non-LAT patients. Likely clinical improvements among LAT patients are believed to be achieved due to proper LAT type recognition and decreased need for physicians' consultations, detoxifications, fewer and shorter hospital admissions and lower drug acquisition costs. The present study is one of the pioneering assessments of health economic justifications for systematic relying on common typologies in clinical practice. These are promising signs of delivering more efficient and cost saving care to alcohol addicts based on LAT in Southeastern Europe. Such evidence should be regarded a stimuli for further research on guidelines impact on the costs of addiction disorders medical care. Applied health economics research in increasingly prevalent addiction disorders could bring partial release to the constrained national health care budgets. Finding alternative ways to deliver cost-effective medical care for alcohol dependents bears particularly heavy policy impact among aging nations of northern hemisphere. Vast economic consequences for the community are particularly evident in these disorders affecting young population. Various substance addictions bring huge opportunity cost due to long lasting decreased working ability, absenteeism and premature mortality. Dissemination of good clinical practice guidelines recommending cost-effective diagnostics and treatment options could be highly effective policy tool for the future of addiction medicine.

## Supporting Information

File S1 (XLSX)

## References

[1] Shield KD, Rylett M, Gmel G, Gmel G, Kehoe-Chan TA, et al (2013) Global alcohol exposure estimates by country, territory and region for 2005–a contribution to the comparative risk assessment for the 2010 Global Burden of Disease Study. Addiction 108: 912–922. 23347092 10.1111/add.12112

[2] Ando B, Rozsa S, Kurgyis E, Szkaliczki A, Demeter I, et al (2013) Direct and Indirect Symptom Severity Indicators of Alcohol Dependence and the Personality Concept of the Biosocial Model. Subst Use Misuse. 10.3109/10826084.2013.841250 24093524

[3] Chisholm D, Rehm J, Van Ommeren M, Monteiro M (2004) Reducing the global burden of hazardous alcohol use: a comparative cost-effectiveness analysis. J Stud Alcohol 65: 782–793. 15700517 10.15288/jsa.2004.65.782

[4] Neighbors CJ, Barnett NP, Rohsenow DJ, Colby SM, Monti PM (2010) Cost-effectiveness of a motivational intervention for alcohol-involved youth in a hospital emergency department. J Stud Alcohol Drugs 71: 384–394. 20409432 10.15288/jsad.2010.71.384 PMC2859787

[5] Wutzke SE, Shiell A, Gomel MK, Conigrave KM (2001) Cost effectiveness of brief interventions for reducing alcohol consumption. Soc Sci Med 52: 863–870. 11234861 10.1016/s0277-9536(00)00189-1

[6] Palmer AJ, Neeser K, Weiss C, Brandt A, Comte S, et al (2000) The long-term cost-effectiveness of improving alcohol abstinence with adjuvant acamprosate. Alcohol Alcohol 35: 478–492. 11022023 10.1093/alcalc/35.5.478

[7] Rychlik R, Siedentop H, Pfeil T, Daniel D (2003) Cost-effectiveness of adjuvant treatment with acamprosate in maintaining abstinence in alcohol dependent patients. Eur Addict Res 9: 59–64. 12644731 10.1159/000068810

[8] Walters D, Connor JP, Feeney GF, Young RM (2009) The cost effectiveness of naltrexone added to cognitive-behavioral therapy in the treatment of alcohol dependence. J Addict Dis 28: 137–144. 19340676 10.1080/10550880902772456

[9] Jakovljevic M, Jovanovic M, Lazic Z, Jakovljevic V, Djukic A, et al (2011) Current efforts and proposals to reduce healthcare costs in Serbia. Ser J Exp Clin Res 12: 161–163.

[10] Ciaranello AL, Perez F, Engelsmann B, Walensky RP, Mushavi A, et al (2013) Cost-effectiveness of World Health Organization 2010 guidelines for prevention of mother-to-child HIV transmission in Zimbabwe. Clin Infect Dis 56: 430–446. 23204035 10.1093/cid/cis858 PMC3540037

[11] Drozda JP (2013) Physician specialty society clinical guidelines and bending the cost curve: comment on “Cost consideration in the clinical guidance documents of physician specialty societies in the United States”. JAMA Intern Med 173: 1097–1099. 23649524 10.1001/jamainternmed.2013.820

[12] French MT, Salome HJ, Sindelar JL, McLellan AT (2002) Benefit-cost analysis of addiction treatment: methodological guidelines and empirical application using the DATCAP and ASI. Health Serv Res 37: 433–455. 12036002 10.1111/1475-6773.031 PMC1430361

[13] Picon PD, Beltrame A, Banta D (2013) National guidelines for high-cost drugs in Brazil: achievements and constraints of an innovative national evidence-based public health policy. Int J Technol Assess Health Care 29: 198–206. 23552016 10.1017/S0266462313000056

[14] Babor TF, Dolinsky ZS, Meyer RE, Hesselbrock M, Hofmann M, et al (1992) Types of alcoholics: concurrent and predictive validity of some common classification schemes. Br J Addict 87: 1415–1431. 1330126 10.1111/j.1360-0443.1992.tb01921.x

[15] Cloninger CR, Bohman M, Sigvardsson S (1981) Inheritance of alcohol abuse. Cross-fostering analysis of adopted men. Arch Gen Psychiatry 38: 861–868. 7259422 10.1001/archpsyc.1981.01780330019001

[16] Lesch WC, Celuch KG (1991) Females' use of alcoholic beverages: a study in context. J Health Soc Policy 2: 23–38. 10116392 10.1300/J045v02n03_02

[17] Lesch OM, Walter H (1996) Subtypes of alcoholism and their role in therapy. Alcohol Alcohol Suppl 1: 63–67. 9845040

[18] Bleich S, Bayerlein K, Reulbach U, Hillemacher T, Bonsch D, et al (2004) Homocysteine levels in patients classified according to Lesch's typology. Alcohol Alcohol 39: 493–498. 15381512 10.1093/alcalc/agh094

[19] Kiefer F, Helwig H, Tarnaske T, Otte C, Jahn H, et al (2005) Pharmacological relapse prevention of alcoholism: clinical predictors of outcome. Eur Addict Res 11: 83–91. 15785069 10.1159/000083037

[20] Leggio L, Kenna GA, Fenton M, Bonenfant E, Swift RM (2009) Typologies of alcohol dependence. From Jellinek to genetics and beyond. Neuropsychol Rev 19: 115–129. 19184441 10.1007/s11065-008-9080-z

[21] Lesch OM, Walter H, Wetschka C, Hesselbrock M, Hesselbrock V (2011) Alcohol and tobacco. Medical and sociological aspects of use, abuse and addiction.Vienna, Austria, Springer-Verlag.

[22] Jakovljevic M, Rankovic A, Rancic N, Jovanovic M, Ivanovic M, et al (2013) Radiology services costs and utilization patterns estimates in Southeastern Europe - a retrospective analysis from Serbia. Value in health regional issues 2: 218–225. 10.1016/j.vhri.2013.07.002 29702868

[23] Jakovljevic MB (2013) Resource allocation strategies in Southeastern European health policy. Eur J Health Econ 14: 153–159. 23143312 10.1007/s10198-012-0439-y

[24] Jovanovic M, Jakovljevic M (2011) Inpatient detoxification procedure and facilities: financing considerations from an Eastern European perspective. Alcohol Alcohol 46: 364–365. 21330296 10.1093/alcalc/agr010

[25] Jakovljevic M, Jovanovic M, Nikic K, Radovanovic A, Pirkovic I, et al (2011) Inpatient detoxification and law enforcement costs following acute drinking event, in typical eastern European upper – middle income, health care setting. Health Behavior & Public Health 1: 1–7.

[26] Pedrero Perez E, Rojo Mota G (2008) [Personality differences between substance addicts and general population. Study of clinical cases with matched controls using Cloninger's TCI-R]. Adicciones 20: 251–261. Spanish. 18818855

[27] Westermeyer J, Specker S (1999) Social resources and social function in comorbid eating and substance disorder: a matched-pairs study. Am J Addict 8: 332–336. 10598216 10.1080/105504999305730

[28] Johnson RS, Tobin JW, Cellucci T (1992) Personality characteristics of cocaine and alcohol abusers: more alike than different. Addict Behav 17: 159–166. 1316717 10.1016/0306-4603(92)90020-v

[29] Westermeyer J, Peng G (1977) Opium and heroin addicts in Laos. II. A study of matched pairs. J Nerv Ment Dis 164: 351–354. 864449 10.1097/00005053-197705000-00007

[30] Stockwell T (1998) Towards guidelines for low-risk drinking: quantifying the short- and long-term costs of hazardous alcohol consumption. Alcohol Clin Exp Res 22: 63S–69S. 9603308

[31] Caputo F, Del Re A, Brambilla R, Grignaschi A, Vignoli T, et al (2014) Sodium oxybate in maintaining alcohol abstinence in alcoholic patients according to Lesch typologies: A pilot study. Journal of Psychopharmacology 28(1): 23–30. 24045881 10.1177/0269881113504015

[32] Djordjevic ND, Jankovic SM (2006) Characteristics of decision-making process during prescribing in general practice. Vojnosanit Pregl 63: 279–285. 16605194 10.2298/vsp0603279d

[33] Rankovic A, Rancic N, Jovanovic M, Ivanovic M, Gajovic O, et al (2013) Impact of imaging diagnostics on the budget–are we spending too much? Vojnosanit Pregl 70: 709–711. 23984624

[34] Jakovljevic M, Vukovic M, Chia-Ching C, Yamada T, Antunovic M, et al (2013) Do policy measures impact cost consciousness of healthcare professionals? Value in Health 16: A542 doi:10.1016/j.jval.2013.08.1376

[35] Haas L, Stargardt T, Schreyoegg J, Schlosser R, Klapp BF, et al (2013) The trade-off between costs and quality of care in the treatment of psychosomatic patients with somatoform pain disorder. Appl Health Econ Health Policy 11: 359–368. 23852985 10.1007/s40258-013-0042-0

[36] Jakovljevic M, Riegler A, Jovanovic M, Djordjevic N, Patek K, et al (2013) Serbian and Austrian alcohol-dependent patients: a comparison of two samples regarding therapeutically relevant clinical features. Alcohol Alcohol 48: 505–508. 23538609 10.1093/alcalc/agt011

[37] Hussey AM, Koch G, Preisser JS, Saville RB (2013) Analysis of matched studies with dichotomous outcomes using nonparametric randomization-based analysis of covariance. Statistics in Biopharmaceutical Research 5.

[38] Zweifel P (2012) The Grossman model after 40 years. Eur J Health Econ 13: 677–682. 22955523 10.1007/s10198-012-0420-9

